# Monsoon-driven short-term temporal changes and geomorphological controls on bacterial community dynamics in Korean coastal lagoons

**DOI:** 10.3389/fmicb.2026.1748786

**Published:** 2026-02-16

**Authors:** Jung Hyun Kwak, Sungmin Hwang, Dongyoung Kim, Dukki Han, Hyun Je Park

**Affiliations:** 1 Jeju Fisheries Research Institute, National Institute of Fisheries Science, Jeju, Republic of Korea; 2 Division of Convergence on Marine Science, Korea Maritime and Ocean University, Busan, Republic of Korea; 3 Department of Marine Ecology and Environment, Gangneung-Wonju National University, Gangneung, Republic of Korea; 4 Department of Marine Bioscience, Gangneung-Wonju National University, Gangneung, Republic of Korea

**Keywords:** 16S rRNA sequencing, bacterial community, coastal wetlands, ecosystem functioning, hydrological variability, restoration

## Abstract

In this study, we investigated monsoon-driven short-term temporal changes in bacterial community composition in two contrasting coastal lagoons in Korea: Gyeongpoho (permanently open) and Hyangho (intermittently open). Using a metabarcoding approach with 16S rRNA gene sequencing, we analyzed the bacterial diversity in these lagoons in relation to environmental variables before and after the monsoon season. Gyeongpoho exhibited greater variability in bacterial composition owing to the continuous seawater exchange, whereas Hyangho freshwater dominance resulted in a more stable microbial community. Salinity fluctuations driven by freshwater inflow significantly influenced microbial diversity, with distinct temporal shifts observed in the dominant bacterial taxa, including Proteobacteria, Bacteroidetes, Acidobacteria, and Chloroflexi. The prevalence of heterotrophic bacteria may be related to their roles in organic matter degradation and nutrient cycling, which are essential for maintaining ecosystem functions. The correlations between bacterial communities and environmental parameters, as revealed by self-organizing map (SOM) and canonical correspondence analysis (CCA) analyses, emphasize the sensitivity of microbial assemblages to hydrological changes. Geomorphological characteristics and hydrological dynamics play important roles in shaping bacterial assemblages. These findings provide crucial insights into the ecological implications of lagoon hydrodynamics and microbial diversity in assessing ecosystem responses to environmental disturbances and climate variability.

## 1 Introduction

Coastal lagoons are common marine ecosystems that account for 13% of coastlines worldwide and generally restrict connections to adjacent oceans through sedimentary barriers (Barnes, 1980; Kjerfve, 1994). These ecosystems are known for being the most productive coastal zones and are highly rich in biodiversity; thus, they are very important for providing a variety of ecological services, including the supply of food and habitat for organisms, climate regulation, hydrological balance, and flood protection (Newton et al., 2018 and references therein). In addition, these areas, as transition zones, are characterized by highly dynamic systems with many biogeochemical processes because of the interactive effects of both terrestrial and marine environmental conditions (Pérez-Ruzafa et al., 2011; Chacón Abarca et al., 2021). Such hydrological and biogeochemical dynamics due to the geomorphological features of coastal lagoons may result in a high degree of spatiotemporal variability in the composition and diversity of natural biological communities as well as local environmental parameters (Carvalho et al., 2005; Garrido et al., 2011). Much scientific attention has been paid to understanding ecological processes through the relationships between environmental factors and assemblages of organisms, providing essential insights for developing effective restoration strategies in coastal ecosystems (Bec et al., 2005; Pitacco et al., 2018; Pérez-Ruzafa et al., 2019).

Bacteria are ubiquitous components of marine environments and play important roles in the biogeochemical cycling of elements and in the production and decomposition of organic matter (Azam et al., 1983; Danovaro and Pusceddu, 2007; Kuchi et al., 2021). Bacterial communities are taxonomically diverse, inhabit a variety of marine habitats, and represent the basis for ecosystem functioning (Ghai et al., 2012; Cadena et al., 2019). In particular, bacterioplankton are highly dynamic in response to monsoon-driven short-term temporal changes in environmental conditions, such as temperature, salinity, pH, and nutrient concentration (Lozupone and Knight, 2007; Jasna et al., 2020; Han et al., 2022). In addition, anthropogenic activities such as ballast water discharge, aquaculture, and seaport-related activity significantly influence bacterial communities in a multifactorial manner (Nogales et al., 2011; Kuchi et al., 2021; da Silva et al., 2022). Considering that estuarine and coastal systems are complex transition zones along environmental gradients with various anthropogenic disturbances, it is essential to study the responses of the composition and functionality of bacterioplankton communities to changing environmental conditions when assessing the synergetic effects of natural and anthropogenic factors on pelagic environments.

Several studies have focused on the dynamics of microbial community composition inhabiting transitional mixed ecosystems of freshwater and seawater based on metabarcoding approaches with the development of molecular techniques (Simon et al., 2014; Gutiérrez-Barral et al., 2021; Zhang et al., 2021). These approaches allow us to describe the metagenomic information of microbial components in relation to altered environmental conditions and/or biogeochemical processes in complex coastal ecosystems on which there is relatively scarce metacommunity data. In this context, the microbial community composition in estuarine and coastal lagoon systems has been reported to spatially shift along a salinity gradient, influenced by interactions between freshwater and seawater (Ghai et al., 2012; Cadena et al., 2019; Zhang et al., 2021; Han et al., 2022). Moreover, these techniques have shown that hydrological changes caused by water flow, tidal fluctuations, freshwater discharge, and heavy precipitation may lead to spatial and temporal variations in microbial diversity and composition in coastal systems (Crump et al., 2004; Wang et al., 2020; Zhao et al., 2020). Although the ecological and biogeochemical effects of variability in physical parameters on microbial diversity and composition have been reported on for various coastal systems, the responses of lagoon communities to such changes in heterogeneous environments have not yet been fully elucidated.

Several lagoons are located along the eastern coastline of the Korean Peninsula, and have been reported to exhibit considerable variability in abiotic and biotic factors owing to their different geomorphic features (Park et al., 2020; Lee et al., 2022). The geomorphology of Korean coastal lagoons can influence the hydrological balance between saline and freshwater masses, resulting in spatiotemporal variations in plant and animal communities (Moon and Lee, 2002; Park et al., 2014; Lee et al., 2015).

In the present study, we aimed to assess the monsoon-driven short-term temporal changes in microbial diversity and composition in coastal lagoons related to the variability in environmental factors by hydrological dynamics between the pre- and post-monsoon seasons. The microbial communities of the water columns of two contrasting lagoons with permanent and intermittent open systems were surveyed using metabarcoding of the 16S rRNA gene. We hypothesize that short-term temporal changes in environmental conditions due to hydrological changes caused by geomorphic characteristics in the lagoons may significantly affect bacterioplankton communities in these lagoons. In the Korean Peninsula, summer monsoon rainfall represents a major hydrological disturbance that alters lagoon connectivity, freshwater inflow, and residence time. The selection of pre- and post-monsoon periods in this study was intended to isolate pulse-driven hydrological effects, including osmotic ecological filtering, nutrient stoichiometry shifts, and terrestrial microbial seeding, which operate on shorter timescales than full seasonal succession. To the best of our knowledge, this is the first study to use the metabarcoding method coupled with next-generation sequencing (NGS) to compare bacterioplankton community compositions and environmental factors at the spatiotemporal scale in Korean coastal lagoons.

## 2 Materials and methods

### 2.1 Study area

**FIGURE 1:**
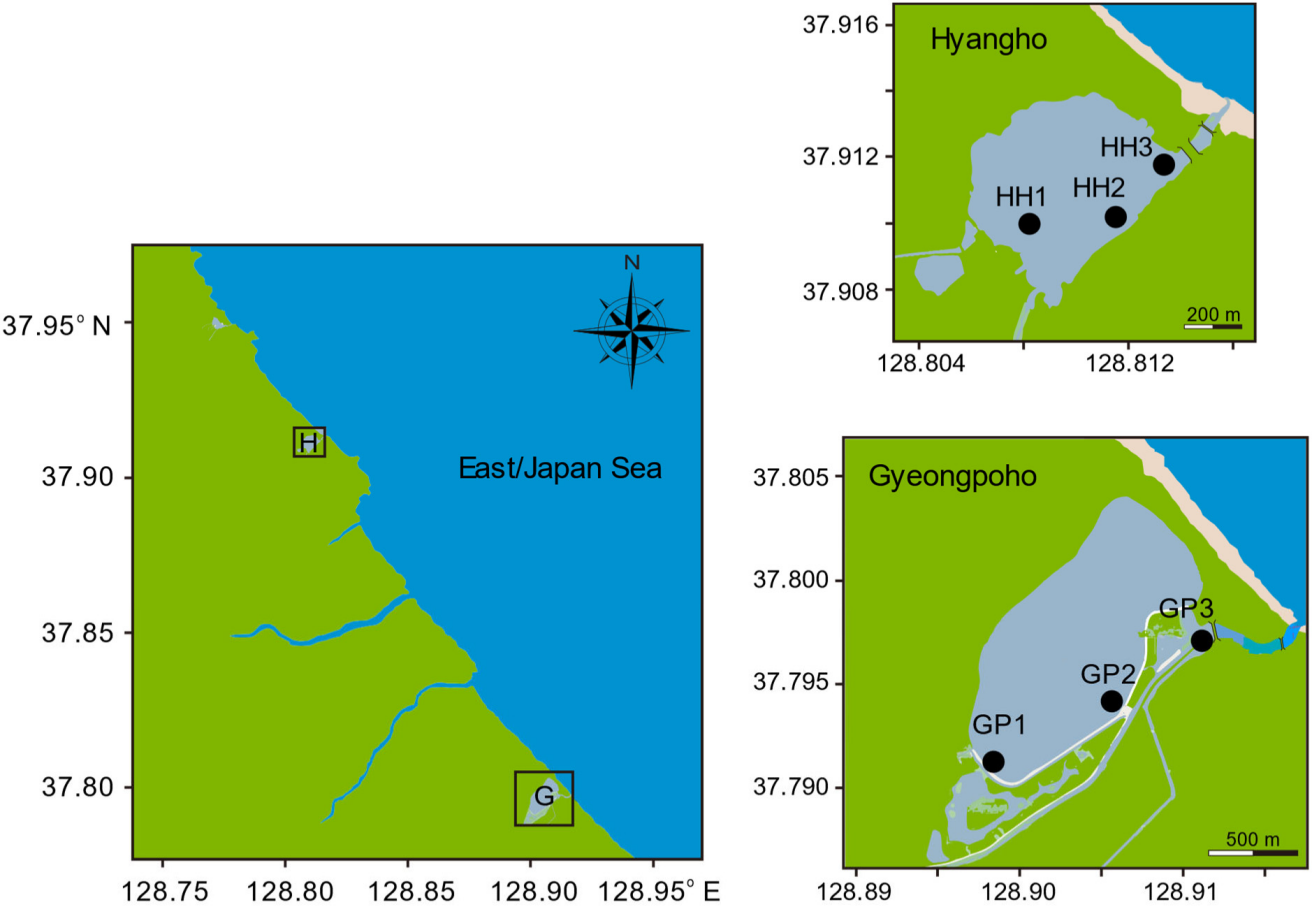
Map of the two coastal lagoons in the eastern coast of the Korean peninsula. Samples (*N* = 6) were collected from three sampling sites in Gyeongpoho and Hyangho, respectively.

The sampling sites were located in two coastal lagoons on the eastern coast of the Korean Peninsula: Gyeongpoho (sites GP1–3) and Hyangho (sites HH1–3), as shown in Figure 1. These lagoons exhibit distinct environmental characteristics, primarily due to the presence or absence of an artificial channel, which influences the salinity gradients of the lagoons, although the depth in both lagoons is generally < 1.0 m. Gyeongpoho, which covers an area of 0.90 km^2^, receives freshwater from several inlets and is connected to the sea by a single artificial channel (WREO, 2008; Yoon et al., 2008). This channel facilitates the continuous exchange of freshwater and seawater, thereby increasing the salinity of the water in the lagoon. Hyangho, which covers an area of 0.14 km^2^, is isolated from the sea by sand dunes. Hyangho is an intermittently open coastal lagoon where heavy rainfall can temporarily modify hydrological conditions; however, during the present sampling period, salinity patterns represent freshwater dominance rather than sustained seawater intrusion. During this period, heavy rainfall creates a temporary channel for the inflow of seawater, thus influencing the salinity of the water in this lagoon (Park et al., 2020).

### 2.2 Sampling and laboratory processing

Sampling was conducted in June and September 2021 at three sites each in Gyeongpoho and Hyangho, which were selected based on their proximity to the sea. Water samples for nutrient, chlorophyll-a (Chl-*a*), and Next-Generation Sequencing (NGS) analyses were collected from the surface at each site. Physicochemical parameters represent single *in situ* measurements per site per sampling month.

The water temperature and salinity were measured using a YSI 30 handheld meter (YSI Inc., Yellow Springs, OH, United States). For Chl-*a* analysis, samples were filtered through 47-mm Whatman GF/F filters. The filtered water samples used for dissolved inorganic nutrient analysis were transferred to acid-washed polyethylene bottles. After pre-filtration through a 200-μm net to remove large debris, samples for NGS analysis were gently filtered through 47-mm diameter polycarbonate filters with a 0.2-μm pore size. These samples were then wrapped in aluminum foil and stored in a deep freezer at –80°C until analysis.

### 2.3 Dissolved inorganic nutrient and Chl-*a*

The dissolved inorganic nutrient samples were analyzed using a QuAAtro nutrient analyser (SEAL Analytical GmbH, Norderstedt, Germany) to determine the concentrations of ammonium (NH_4_), nitrate (NO_3_), nitrate (NO_2_), phosphate (PO_4_), and silicate (SiO_4_). The procedures followed for automated chemical analysis were in accordance with established methods, with detection limits of 0.01 μM for NH_4_, 0.1 μM for NO_3_, 0.1 μM for NO_2_, 0.02 μM for PO_4_, and 0.1 μM for SiO_4_ (Murphy and Riley, 1962; Helder and De Vries, 1979; Hansen and Koroleff, 1999). For Chl-*a* analysis, filter samples were extracted with 90% acetone in the dark at 4°C for 20 h. The concentrations of Chl-*a* were then determined fluorometrically using a fluorometer (Turner Designs, Sunnyvale, CA, United States) according to the method described by Parsons et al. (1984).

### 2.4 PCR amplicon construction and sequencing

For NGS analysis we used duplicate filtered samples. Genomic DNA was extracted from the filters using a PowerWater DNA Isolation Kit (Mo Bio Laboratories, Carlsbad, CA, United States). For bacterial community analysis, DNA was extracted from duplicate 0.2-μm filtered water samples at each site, and sequence data were rarefied to 10,000 reads per sample prior to diversity analyses. The DNA concentration was measured using Quant-iT PicoGreen dsDNA Reagent (Molecular Probes, Eugene, OR, United States). To sequence the bacterial 16S rRNA genes targeting the V34 region, we utilized the Illumina MiSeq platform (Macrogen, Seoul, South Korea), following a two-step PCR process involving both amplicon and index PCR. The PCR conditions and primers were prepared according to the library preparation guidelines for the Illumina MiSeq platform. Initial PCR reactions were conducted in triplicate using the KAPA HiFi Hotstart ReadyMix PCR kit (KAPA BioSystems, Wilmington, MA, United States) with Illumina amplicon primers. The resulting PCR amplicons were purified using the Qiaquick PCR Purification Kit (Qiagen, Valencia, CA, United States), and subsequent index PCRs were performed in accordance with protocol specified by Illumina. The indexed PCR products were quantified using a Qubit 2.0 Fluorometer (Invitrogen, Carlsbad, CA, United States). Finally, the purified amplicons were pooled in equimolar amounts and sequenced. The metabarcoding sequences from this study were deposited in the NCBI Sequence Read Archive (SRA) under accession number PRJNA1251985.

### 2.5 Data analysis

Sequencing data were analyzed to investigate bacterial diversity and community composition using the Mothur software package (v. 1.40.5) (Schloss et al., 2009). A total of 240,000 sequences were obtained from duplicate samples after processing the sequencing data as previously described (Han et al., 2024). Briefly, sequencing data were processed using the Mothur SOP (Kozich et al., 2013), including error correction, removal of nonbacterial sequences, and normalization to 10,000 sequences per sample. The filtered sequences were clustered into amplicon sequence variants (ASVs) to analyze bacterial alpha and beta diversity. Chao1 and ACE indices were used for alpha diversity, while thetayc dissimilarity calculator in Mothur and non-metric multidimensional scaling (NMDS) were applied to assess beta diversity. Differences in community composition were further evaluated using analysis of molecular variance (AMOVA). The bacterial community composition was determined using the SILVA database (Silva_v138). After bacterial taxonomic classification, the major bacterial populations (> 1% of the total sequences) were selected for further taxonomic analyses. The relative abundances of the major 12 bacterial populations (phylum level) at the study sites were determined using a self-organizing map (SOM) algorithm, a heuristic model for visualizing linear and nonlinear relationships in high-dimensional datasets (Kohonen, 1982). During the SOM training process, data were assigned to neurons in the input layer. This process involves coarse training and fine-tuning, culminating in the formation of an output layer with output neurons. In this layer, a real sample was allocated to the neuron that most closely resembled it, and samples with similar characteristics were grouped into adjacent neurons on a two-dimensional grid. The number of neurons is typically determined using the heuristic rule of 5√n, or by considering quantisation (QE) and topographic (TE) error samples (Vesanto and Alhoniemi, 2000; Park et al., 2003; Park et al., 2006). However, Céréghino et al. (2001) noted the absence of a theoretical principle to determine the optimum map size. Despite these guidelines, we chose a smaller map because of the presence of numerous empty output neurons. In this study, a 3 × 2 grid of output neurons was used to cluster the physicochemical data (13 variables and 12 samples). Post-training, cluster boundaries between different SOM units were identified using Ward’s linkage method and Euclidean distance measurement. The SOM training and clustering procedures were executed using R version 4.1.1 (Licen et al., 2021).

Canonical Correspondence Analysis (CCA) was conducted using the CANOCO program to delineate the relationships between environmental factors (eight variables) and bacterial species (13 variables) at each study site. Canonical correspondence analysis (CCA) was used as an exploratory ordination tool. Permutation-based significance testing was not performed due to the limited sample size (*N* = 12), and results were interpreted cautiously. This method, as described by Ter Braak (1986) and Sundbäck and Snoeijs (1991), effectively illustrates the associations between multiple environmental variables and species distribution.

## 3 Results

### 3.1 Physicochemical factors

The measured values of temperature, salinity, NH_4_, NO_2_, NO_3_, PO_4_, SiO_4_, and Chl-*a* for all sites are presented in Table 1. During the study period, water temperature ranged from 19.3 to 25.3°C and salinity from 6.5 to 20.5 in Gyeongpoho, while in Hyangho, water temperature ranged from 24.3 to 26.6°C and salinity from 4.4 to 5.5. In Gyeongpoho, the highest water temperatures were observed at GP1 in both June and September, which was associated with high freshwater inflows, whereas salinity varied across at least five of the sites. In Hyangho, the highest water temperatures were recorded at HH1 in both months, with salinity differences between the sites within 0.7. Concentrations of NH_4_, NO_2_, NO_3_, PO_4_, SiO_4_, and Chl-*a* in Gyeongpoho ranged from 0.5 to 25.4 μM, 0.0 to 2.3 μM, 0.6 to 13.6 μM, 0.7 to 2.1 μM, 4.7 to 53.4 μM, and 0.7 to 39.5 μg l^–1^, respectively. In Hyangho, these concentrations ranged from 0.1 to 6.2 μM, 0.1 to 1.0 μM, 0.8 to 5.9 μM, 0.2 to 0.9 μM, 11.7 to 59.8 μM, and 9.0 to 20.9 μg l^–1^, respectively. In Gyeongpoho, the NH_4_, NO_3_, and PO_4_ concentrations were notably high at GP3 in both months, whereas the Chl-*a* concentrations were significantly lower. In Hyangho, nutrient and Chl-*a* concentrations were the highest at HH1 in both months.

**TABLE 1:** Environmental parameters—including temperature, salinity, and concentrations of ammonium (NH_4_), nitrite (NO_2_), nitrate (NO_3_), phosphate (PO_4_), silicate [Si(OH)_4_], and chlorophyll-*a* (Chl-*a*).

Location	Station	Date	Code	Temperature	Salinity	NH_4_^+^	NO_2_^–^	NO_3_	PO_4_	SiO_4_	Chl-*a*
				(°C)		(μ M)	(μ M)	(μ M)	(μ M)	(μ M)	(μ g l^–1^)
Gyeongpoho	GP1	June 2022	6GP1	23.3	6.5	8.5	0.7	4.9	0.8	39.6	29.7
	GP2	June 2022	6GP2	22.0	20.0	4.6	1.2	4.6	0.7	53.4	39.5
	GP3	June 2022	6GP3	19.3	20.5	25.3	2.3	12.6	1.9	32.4	0.7
	GP1	September 2022	9GP1	25.3	12.9	0.9	0.2	1.0	1.3	27.7	20.9
	GP2	September 2022	9GP2	25.2	18.1	0.5	0.0	0.6	1.3	4.7	25.9
	GP3	September 2022	9GP3	24.4	17.9	25.4	2.2	13.6	2.1	17.0	4.3
Hyangho	HH1	June 2022	6HH1	26.0	5.0	6.2	1.0	5.9	0.5	30.8	13.6
	HH2	June 2022	6HH2	24.6	5.4	0.1	0.2	2.7	0.2	11.7	9.0
	HH3	June 2022	6HH3	24.3	5.5	0.1	0.2	2.2	0.3	22.0	11.6
	HH1	September 2022	9HH1	26.6	4.4	1.1	0.1	1.4	0.9	59.8	20.9
	HH2	September 2022	9HH2	24.5	4.4	0.4	0.1	0.8	0.4	54.6	13.2
	HH3	September 2022	9HH3	25.5	5.1	0.6	0.1	0.8	0.4	50.4	12.5

### 3.2 Bacterial community composition

**FIGURE 2:**
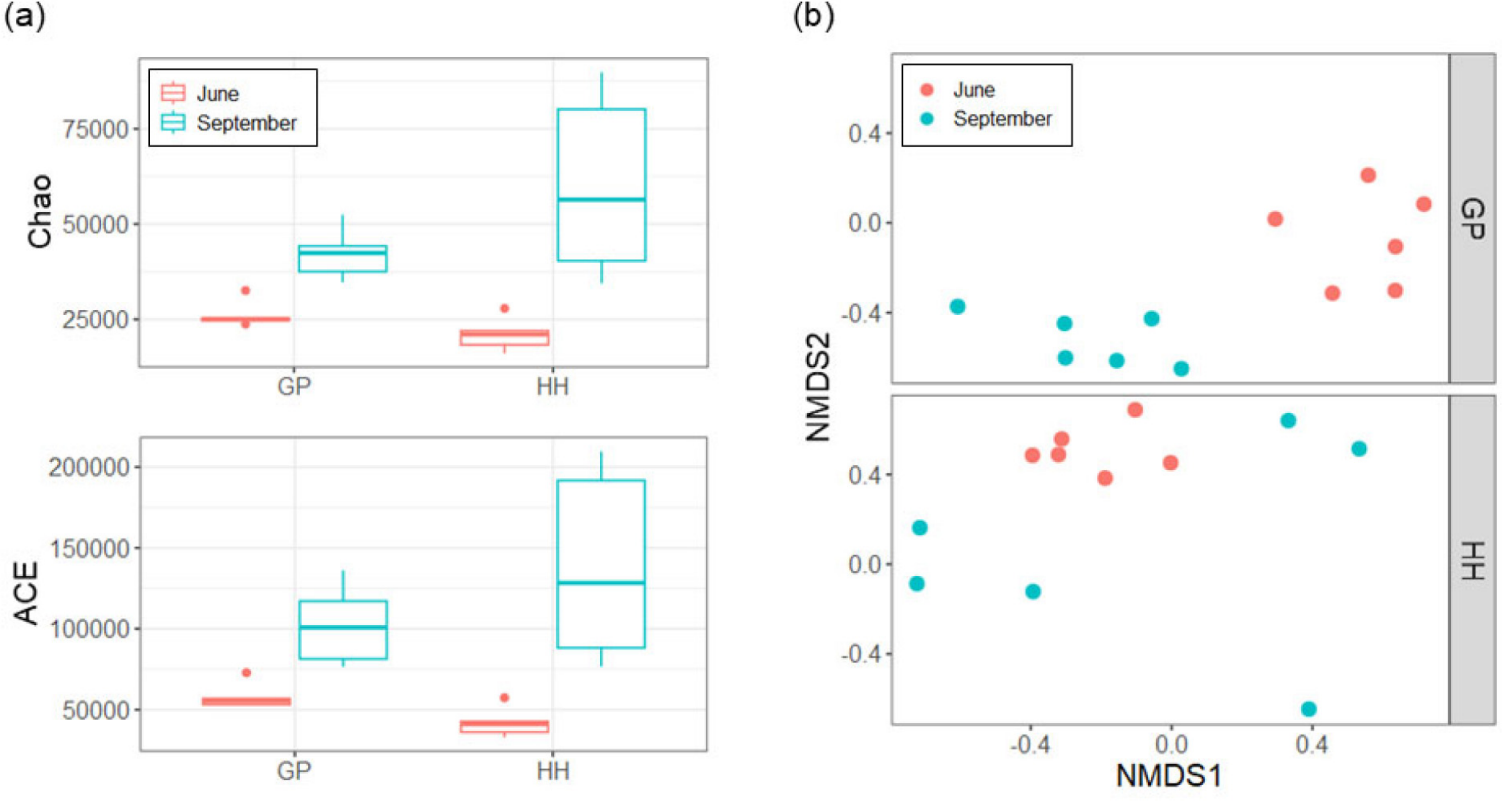
Bacterial diversity analysis. Alpha diversity patterns with Chao and ACE **(a)**. Beta diversity on NMDS **(b)**. The stress value of the two-dimensional solution on the NMDS was approximately 0.35. Differences in bacterial community composition were statistically supported by AMOVA, showing significant differences between sampling periods (June vs. September, *p* < 0.001) and between lagoons (Gyeongpoho vs. Hyangho, *p* < 0.001).

**FIGURE 3:**
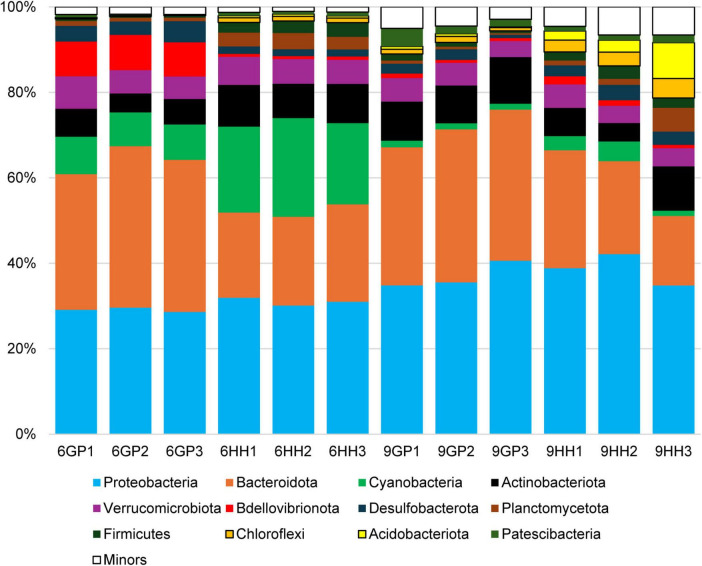
Bacterial community composition at phylum level. Minors (<1%) represent the sum of rare phylotypes (genetically distinct types) that make up < 1% of the total sequences.

The bacterial sequences (*N* = 240,000) were clustered at 108,856 ASVs. ASVs were analyzed to assess both alpha and beta diversity in the bacterial communities (Figure 2). Regarding alpha diversity, the Chao1 and ACE indices showed similar patterns in both Gyeongpoho and Hyangho, with higher species richness in September than in June. In terms of beta diversity, a clear distinction was observed between the bacterial communities in September and June at both Gyeongpoho and Hyangho, which was statistically supported by AMOVA (*p* < 0.001). In this study, the sequences were classified at the phylum level using a taxonomic database (Figure 3). Proteobacteria and Bacteroidetes comprised the majority of these sequences, consistent with their recognition as dominant bacterial populations in coastal estuaries, particularly in Korea (Han et al., 2020; Han et al., 2022; Seo et al., 2017). Visualization of the monsoon-driven short-term temporal changes across all samples revealed that heterotrophic bacteria, specifically Proteobacteria and Bacteroidetes, were dominant in all samples. In contrast, photosynthetic cyanobacteria were more abundant in the June samples than in the September samples, for both Gyeongpoho and Hyangho. Among the dominant heterotrophic bacteria, Proteobacteria maintained a relatively constant distribution across all samples, whereas Bacteroidetes exhibited greater variability. Beta-diversity dispersion analysis showed greater variability in bacterial community composition in Gyeongpoho, consistent with its broader salinity range and continuous seawater exchange. Notably, sequences assigned to Bdellovibrionota were particularly enriched in June samples from Gyeongpoho compared to the other samples. In contrast, Acidobacteriota and Chloroflexi sequences were more prevalent in September samples from Hyangho.

### 3.3 Self-organizing map and canonical correspondence analysis

**FIGURE 4:**
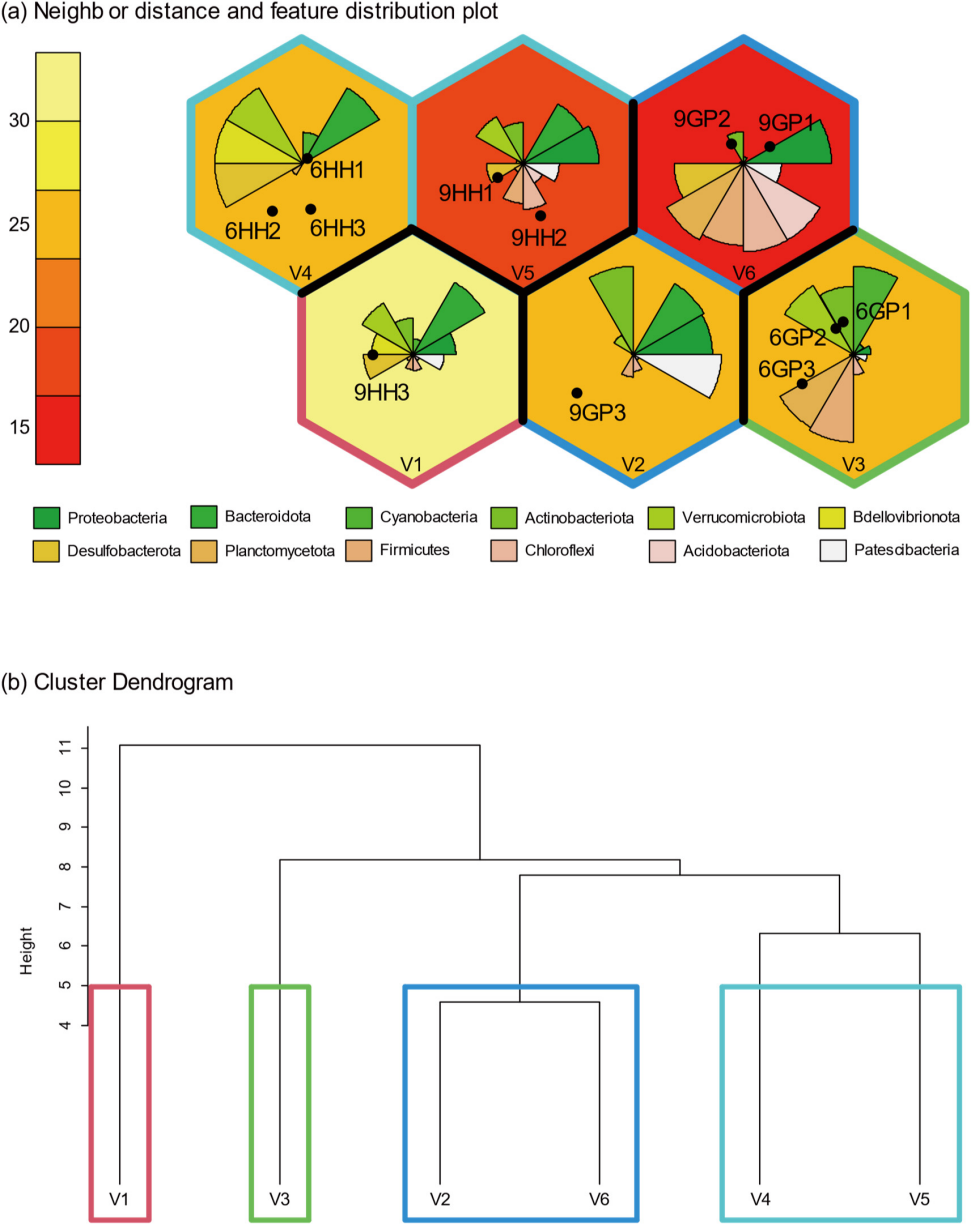
Grouping of characteristics according to time and site on the Kohonen self-organizing map algorithm (SOM) trained with the top 12 OTUs **(a)**. Gradient of unit box color (white-yellow) represents neighbor distance. Dendrogram of the trained SOM groups based on Ward’s linkage method **(b)**.

The SOM training results were condensed onto 3 ×2 rectangular grid maps based on 12 datasets (6 sites over 2 months) of 13 bacteria in Gyeongpoho and Hyangho (Figure 4). The cluster dendrograms of the SOM results grouped major four clusters coinciding with the study area and month. These bacterial clusters represent distinct environmental conditions for sites within Gyeongpoho and Hyangho, with noticeable variations in their parameters.

**FIGURE 5:**
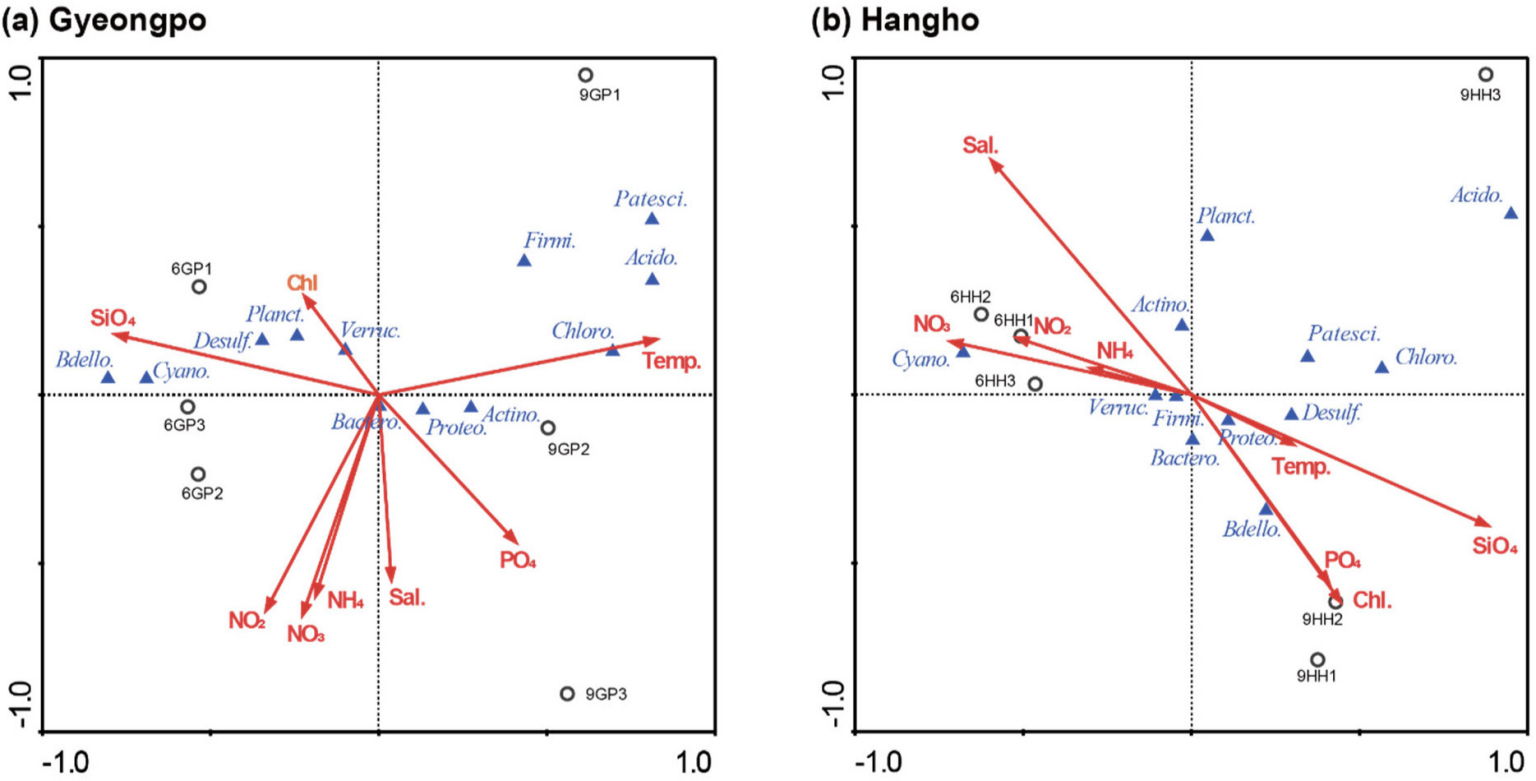
Ordination diagram obtained from canonical correspondence analysis based on the top 12 OTUs located in Gyungpo **(a)**, and Hangho **(b)** with respect to, temperature, salinity nutrients (nitrate, nitrite, ammonium, phosphate, and silicate), and Chl-*a*.

Canonical correspondence analysis (CCA) was used to investigate the relationships between environmental factors (water temperature, salinity, nutrients, and Chl-*a*) and bacterial community composition (Figure 5 and Table 2). The first two ordination axes of the CCA explained 96.6 and 95.9% of the species-environment relationships in Gyeongpoho and Hyangho, respectively. Samples from each dataset, represented by open circles, are dispersed in the CCA diagrams according to the characteristics of the environmental factors and bacterial species. The samples were closely distributed and corresponded to the clusters classified in the SOM analysis based on axis 1. In Gyeongpoho, temperature and PO_4_ were positioned opposite to NH_4_, NO_2_, NO_3_, and SiO_4_ along axis 1 of the biplot, reflecting a negative correlation, whereas salinity and Chl-*a* were weakly related to other environmental factors. In Hyangho, salinity, NH_4_, NO_2_, and NO_3_ were negatively correlated with PO_4_, SiO_4_ and Chl-*a*, whereas temperature was only weakly correlated with other environmental factors. The positions of the bacterial species in the biplots were widely distributed, reflecting their correlation with environmental factors.

**TABLE 2:** Summary of the results from canonical correspondence analysis.

Gyeongpoho	Axis 1	Axis 2	Axis 3	Axis 4	Total inertia
Eigenvalues	0.095	0.002	0.002	0.001	0.100
Species-environment correlations	1.000	1.000	1.000	1.000	
Cumulative percentage variance					
—Of species data	94.6	96.6	98.3	99.6	
—Of species environment relation	94.6	96.6	98.3	99.6	
Sum of all unconstrained eigenvalues					0.100
Sum of all canonical eigenvalues					0.100
Hyangho	Axis 1	Axis 2	Axis 3	Axis 4	Total inertia
Eigenvalues	0.089	0.036	0.003	0.002	0.130
Species-environment correlations	1.000	1.000	1.000	1.000	
Cumulative percentage variance					
—Of species data	68.5	95.9	98.4	99.6	
—Of species environment relation	68.6	95.9	98.4	99.6	
Sum of all unconstrained eigenvalues					0.225
Sum of all canonical eigenvalues					0.131

## 4 Discussion

This study assessed monsoon-driven short-term temporal changes in the characteristics of environmental conditions and bacterial communities in two contrasting coastal lagoons (permanently and intermittently open) in Korea. The major finding was the identification of differences in the seasonal variations in bacterial communities in relation to environmental changes observed in the two types of lagoons. These results highlight the ecological importance of different bacterial communities in response to hydrological changes related to freshwater input between the two systems after a monsoonal event. Several studies have reported that salinity change due to freshwater intrusion is one of the most important factors controlling spatiotemporal variations in bacterial communities in estuarine and coastal systems (Ghai et al., 2012; Li et al., 2017; Ozbayram et al., 2024). Overall, the seasonal hydrological differences caused by the geomorphological characteristics of the two lagoons may lead to considerable spatiotemporal variations in the bacterial communities, which are expected to contribute to the dynamics of the biogeochemical cycles of the lagoons.

The present study found spatiotemporal variations in the bacterial communities of coastal lagoon systems despite similar seasonal tendencies at the Gyeongpoho and Hyangho sites. In general, bacterial communities in aquatic ecosystems, including lagoons, are characterized by spatial and temporal dynamics due to the high variability in environmental factors (Jasna et al., 2020; Han et al., 2022). In particular, differences in environmental conditions caused by the different hydrodynamic regimes of Gyeongpoho and Hyangho lagoons may lead to differences in the biogeochemical processes of suspended particulate organic matter (Lee et al., 2022). Such spatiotemporal variability is influenced by the geomorphological characteristics of the two systems, which can bring about changes in the hydrological conditions through the input of freshwater and seawater to the lagoons (Kjerfve, 1994). The mixture of organic matter present in coastal lagoons is highly heterogeneous and is composed of living micro/macroalgae and detritus from terrestrial and marsh materials (García-Ayllón, 2017). These differences in organic matter between Gyeongpoho and Hyangho may reflect monsoon-driven short-term temporal changes in dominant heterotrophic bacteria. Thus, such a difference in bacterial community composition between the lagoons suggests a relationship between the difference in freshwater- and seawater-dominated waters by geomorphological connectivity with the adjacent coast between the permanently and intermittently open lagoons. Several studies have reported that the degree of connection of the lagoon to the ocean by geomorphological features can lead to significant changes in microbial composition and diversity owing to seasonal and spatial variations in the origin of organic or inorganic matter (Laque et al., 2010; Boadella et al., 2021; Trombetta et al., 2022).

The dominance of heterotrophic bacteria in Gyeongpoho and Hyangho highlights their crucial roles in nutrient cycling, particularly in the degradation of organic matter (Nagata, 2008). In contrast to the consistent dominance of heterotrophic bacteria, photosynthetic cyanobacteria displayed seasonal variation, with a predominance in summer compared with fall in both Gyeongpoho and Hyangho. This seasonal increase is likely driven by greater sunlight availability and elevated nutrient concentrations during summer, which create favorable conditions for cyanobacterial growth (Pruvost et al., 2015). In this study, Proteobacteria exhibited a relatively stable distribution across all samples, whereas Bacteroidetes showed greater variability. The stability of Proteobacteria can be attributed to their adaptability to a wide range of environmental conditions and ability to utilize diverse organic substrates (Nagata, 2008). This resilience ensures their consistent presence, regardless of seasonal or spatial changes. However, the variability in the distribution of Bacteroidetes may reflect their reliance on specific organic substrates or environmental conditions that fluctuate over time and space. This suggests that Bacteroidetes populations are more responsive to changes in organic matter availability and other dynamic environmental factors, potentially influencing their roles in carbon cycling and organic matter degradation in coastal lagoon ecosystems. Although direct measurements of DOC, POC, and C:N ratios were not available, the dominance of heterotrophic taxa likely reflects potential metabolic capacity rather than quantified organic matter degradation rates. A comparison of the microbial communities in Gyeongpoho and Hyangho revealed distinct spatial patterns in bacterial composition. The unique enrichment of Bdellovibrionota in Gyeongpoho and Acidobacteriota and Chloroflexi in Hyangho suggests that local environmental conditions play a significant role in shaping microbial community structures. Bdellovibrionota are well known as obligate predators of Gram-negative bacteria, and their increased relative abundance in Gyeongpoho during June likely reflects a high availability of heterotrophic bacterial prey. This pattern suggests an intensified microbial trophic interaction, potentially associated with elevated organic matter and post-bloom conditions during the pre-monsoon period, rather than a simple response to physicochemical environmental factors. Monsoon-driven freshwater inputs can restructure lagoon bacterioplankton through multiple interacting mechanisms, including osmotic stress imposed by salinity depression, altered nutrient stoichiometry, increased availability of terrestrially derived organic substrates, and watershed-associated microbial loading. The enrichment of Acidobacteriota and Chloroflexi in Hyangho during September is therefore interpreted as reflecting soil runoff influence rather than intrinsic lagoon biogeochemical differentiation.

The results of the SOM analysis revealed a distinct clustering pattern of bacterial communities according to both the sampling period (June vs. September) and lagoon type (Gyeongpoho vs. Hyangho). Although SOM were applied to a relatively small dataset (*N* = 12), the method was used as an exploratory visualization tool to identify non-linear associations between bacterial communities and environmental gradients. SOM topology was heuristically constrained, and resulting clusters were cross-validated against Ward’s hierarchical clustering, indicating that SOM results primarily reflect underlying distance-based structure rather than independent overfitting. This finding highlights the significance of seasonal hydrological changes and geomorphological characteristics in determining the spatiotemporal dynamics of microbial communities in coastal lagoons (Han et al., 2022). Gyeongpoho, with its wider salinity range due to continuous seawater exchange, exhibited greater variability in bacterial community composition across the sites. In contrast, Hyangho, which is predominantly freshwater-dominated, showed less temporal variability but a higher abundance of specific taxa, such as Acidobacteriota and Chloroflexi in September. CCA further elucidated the relationship between environmental parameters and bacterial community structure. In Gyeongpoho, temperature and PO_4_ were inversely associated with NH_4_, NO_2_, NO_3_, and SiO_4_, indicating distinct environmental conditions during the study period. Salinity and Chl-*a* were weakly correlated with other environmental variables, suggesting a complex interplay between physical and biological factors, such as water temperature, nutrients, and the euryhaline characteristics of bacterial assemblages, in structuring bacterial assemblages (Ghai et al., 2012). In Hyangho, PO_4_, SiO_4_, and Chl-*a* were negatively correlated with salinity, indicating that changes in water quality driven by freshwater dominance rather than seawater intrusion may significantly affect phytoplankton biomass and bacterial community structure (Crump et al., 2004). The observed bacterial community shifts in Hyangho are more plausibly explained by freshwater-driven ecological filtering rather than marine intrusion, as salinity decreased from June to September. The positioning of the bacterial taxa in the CCA biplots demonstrated strong correlations with these environmental variations, supporting the hypothesis that hydrological variability drives microbial community shifts. Together, the SOM and CCA results provide complementary insights into the spatiotemporal structuring of bacterial communities. The clustering patterns and environmental correlations observed in both analyses underscore the importance of lagoon geomorphology and seasonal rainfall in modulating microbial dynamics. The permanently open system (Gyeongpoho) was more influenced by broader environmental gradients induced by marine-freshwater mixing, whereas the intermittently open system (Hyangho) was characterized by more stable freshwater conditions and a greater influence of seawater inflow. These findings suggest that the amount of freshwater and marine water inflow, along with the degree of hydrological connectivity, are crucial factors shaping the bacterial community composition and, consequently, biogeochemical processes in temperate coastal lagoons (Nogales et al., 2011; Zhao et al., 2020). This study represents a snapshot comparison between pre- and post-monsoon conditions and does not capture recovery dynamics, winter microbial regimes, or long-term seasonal succession. In addition, the limited number of sampling stations per lagoon may not fully resolve fine-scale spatial heterogeneity at freshwater–seawater mixing zones.

## 5 Conclusion

This study highlights the significant role of seasonal hydrological variations and geomorphological characteristics in shaping bacterial community structures in two contrasting coastal lagoons (the permanently open Gyeongpoho and intermittently open Hyangho open lagoons). The observed monsoon-driven short-term temporal changes in bacterial communities suggest the ecological importance of hydrological connectivity and freshwater-seawater interactions in coastal lagoon ecosystems. These findings demonstrate that salinity fluctuations driven by freshwater inflow after monsoon events are important factors that influence bacterial diversity and composition. More dynamic seawater exchange in Gyeongpoho resulted in a higher degree of bacterial variability, whereas the relatively stable conditions in Hyangho promoted specific microbial assemblages adapted to freshwater dominance. These differences contribute to distinct biogeochemical processes, particularly organic matter degradation and nutrient cycling, emphasizing the role of microbial communities in sustaining lagoon ecosystem functions. The predominance of heterotrophic bacteria in both lagoons highlights their essential roles in organic matter decomposition and carbon cycling, which can influence trophic interactions and water quality. Furthermore, the correlations between environmental parameters and bacterial community structures, as revealed by SOM and CCA, enhance the ecological perspective that microbial assemblages serve as sensitive indicators of environmental changes. Our findings demonstrate that bacterial community structure in coastal lagoons is shaped by the interaction between monsoon-driven short-term freshwater disturbance and geomorphology-dependent spatial species sorting. While this study provides insight into disturbance-driven ecological filtering, higher-frequency temporal sampling will be required to resolve recovery processes and long-term microbial succession.

## Publihser’s note

All claims expressed in this article are solely those of the authors and do not necessarily represent those of their affiliated organizations, or those of the publisher, the editors and the reviewers. Any product that may be evaluated in this article, or claim that may be made by its manufacturer, is not guaranteed or endorsed by the publisher.

## Data Availability

The data presented in this study are publicly available. The data can be found at: https://www.ncbi.nlm.nih.gov, accession PRJNA1251985.
